# Risk Factors for the In-Hospital Mortality in Pyogenic Vertebral Osteomyelitis: A Cross-Sectional Study on 9753 Patients

**DOI:** 10.3390/jcm12144805

**Published:** 2023-07-21

**Authors:** Tomasz Piotr Ziarko, Nike Walter, Melanie Schindler, Volker Alt, Markus Rupp, Siegmund Lang

**Affiliations:** 1 Department for Trauma Surgery, University Hospital Regensburg, 93053 Regensburg, Germany; 2 Department for Psychosomatic Medicine, University Hospital Regensburg, 93053 Regensburg, Germany

**Keywords:** pyogenic vertebral osteomyelitis, mortality, risk-factors

## Abstract

Background: Pyogenic vertebral osteomyelitis represents a clinical challenge associated with significant morbidity and mortality. The aim of this study was to analyze potential risk factors for the in-hospital mortality of vertebral osteomyelitis (VO) patients. Methods: Based on the International Classification of Diseases, 10th Revision (ICD-10) codes for VO (“M46.2-”, “M46.3-”, and “M46.4-”) data for total case numbers, secondary diagnoses, and numbers of in-hospital deaths were extracted from the Institute for the Hospital Remuneration System (InEK GmbH). Odds ratios (OR) for death were calculated for several secondary diseases and factors of interest. Results: Despite age, certain comorbidities were found to be strongly associated with increased mortality risk: Heart failure (OR = 2.80; 95% CI 2.45 to 3.20; *p* < 0.01), chronic kidney disease (OR = 1.83; 95% CI 1.57 to 2.13; *p* < 0.01), and diabetes with complications (OR = 1.86; 95% CI 1.46 to 2.38; *p* < 0.01). Among the complications, acute liver failure showed the highest risk for in-hospital mortality (OR = 42.41; 95% CI 23.47 to 76.62; *p* < 0.01). Additionally, stage III kidney failure (OR = 9.81; 95% CI 7.96 to 12.08; *p* < 0.01), sepsis (OR = 5.94; 95% CI 5.02 to 7.03; *p* < 0.01), acute respiratory failure (OR = 5.31; 95% CI 4.61 to 6.12; *p* < 0.01), and systemic inflammatory response syndrome (SIRS) (OR = 5.19; 95% CI 3.69 to 5.19; *p* < 0.01) were associated with in-hospital mortality. When analyzing the influence of pathogens, documented infection with *Pseudomonas aeruginosa* had the highest risk for mortality (OR = 2.74; 95% CI 2.07 to 3.63; *p* < 0.01), followed by Streptococci, *Escherichia coli*, and *Staphylococcus aureus* infections. Conclusions: An early assessment of individual patient risk factors may be beneficial in the care and treatment of VO to help reduce the risks of mortality. These findings emphasize the importance of closely monitoring VO patients with chronic organ diseases, early detection and treatment of sepsis, and tailored empirical antibiotic therapy. The identification of specific pathogens and antibiotic susceptibility testing should be prioritized to improve patient outcomes in this high-risk population.

## 1. Introduction

Musculoskeletal infections present a significant challenge in the fields of orthopedics and trauma surgery [1]. When infections of the spine occur without prior surgery or implanted devices, they are known as pyogenic vertebral osteomyelitis (VO), also referred to as spinal osteomyelitis or spondylodiscitis [2]. Hospitalization is often necessary for patients with VO [3]. Delayed diagnosis is common, particularly in cases involving low-virulence pathogens, and this can lead to high morbidity and mortality rates [4]. It is crucial to highlight the increasing importance of infections caused by coagulase-negative staphylococci (CONS) in this context [5,6,7]. Epidemiological studies have consistently reported a rise in the incidence of VO, indicating an ongoing challenge for healthcare systems [8,9,10]. Additionally, the treatment of elderly patients continues to be of great importance to musculoskeletal surgeons [11]. This trend can be attributed to the increasing number of comorbidities in an aging population, as well as advancements in imaging techniques that have improved diagnostics and resulted in a higher number of documented VO cases [12,13,14,15]. Furthermore, the standardization of pathogen identification methods has contributed to the detection of VO [16]. Recent publications have provided updated diagnostic and therapeutic approaches to VO treatment [17,18]. However, a significant heterogeneity remains in reports on the epidemiology of VO, with limited analyses of nationwide databases and an incomplete understanding of the risk factors associated with in-hospital mortality [8,10,19].

Therefore, this study aimed to analyze potential risk factors for in-hospital mortality among VO patients.

## 2. Materials and Methods

### 2.1. Data Source

In accordance with section 17b of the German Hospital Financing Act (KHG), a universal and performance-based remuneration system has been established for the provision of general hospital services. This includes a flat-rate compensation approach based on the German Diagnosis Related Groups system (G-DRG system). Each inpatient treatment case is thus remunerated via a corresponding DRG lump sum payment, providing standardized compensation aimed at maintaining uniformity across the healthcare system.

The Institute for the Remuneration System in Hospitals (InEK GmbH) serves as a substantial data source, providing in-depth data on primary and secondary diagnoses, all classified using the International Classification of Diseases, 10th Revision (ICD-10) system. Moreover, it presents data regarding patient demographics such as age and gender, as well as reasons for discharge, including mortality-related circumstances.

The InEK DatenBrowser facilitates a comprehensive analysis of these data, offering a cross-sectional view of healthcare trends and outcomes, and it includes information extending back to the year 2019.

Pre- and post-stationary services are included in the datasets as per § 1 para. 6 FPV. In the remuneration area “PSY”, cases with equivalent inpatient psychiatric treatment are also included in the base population.

For cases with partial inpatient care in the remuneration area “DRG”, counting is based on the number of transmitted data records, not adhering to the provisions of § 9 FPV where cases with regular or multiple treatments per quarter are counted as one case. This may result in a higher or lower case number compared to the number of delivered data records, and evaluation results for partially inpatient care must be interpreted considering these limitations.

For the purposes of this study, an analysis was conducted specifically for the year 2020. The focus was on the ICD-10 codes for vertebral osteomyelitis (VO)—“M46.2-”, “M46.3-”, and “M46.4-”. The extracted data encompassed total case numbers, secondary diagnoses coding for comorbidities and complications, and instances of in-hospital deaths related to VO. This enabled an insightful exploration of the prevalence and outcomes associated with this specific condition. To ensure relevance, we chose to exclusively report on conditions that accounted for a minimum of 0.5% of the total cases by default.

### 2.2. Statistical Analysis

Statistical analysis was carried out using SPSS software version 28 (SPSS Inc, Chicago, IL, USA). The frequencies of secondary diagnoses, identified through coded data, are represented both as absolute numbers and as proportions of the total cases. To enhance interpretability, these diagnoses were segregated into two distinct categories: comorbidities and complications. Separately, coded data pertaining to pathogens were scrutinized.

In this study, univariate analysis was utilized to individually analyze each variable for its potential as a risk factor associated with in-hospital mortality among patients diagnosed with vertebral osteomyelitis (VO) in the year 2020. The dataset used for this examination included a wide spectrum of patient outcomes, including both cases with and without documented in-hospital deaths.

The statistical strength of the association between exposure to certain factors and the occurrence of the defined outcome—in-hospital mortality, in this case—was quantified using odds ratios (OR). An OR < 1.0 signified a negative association, indicating that the exposure to the variable under consideration was associated with lower odds of in-hospital mortality. Conversely, an OR > 1.0 represented a positive association, implying that the exposure was linked with higher odds of the defined outcome. ORs were calculated for several comorbidities and complications of interest. To complement these ORs, lower and upper 95% Confidence Intervals (CI) were also derived, furnishing an estimate of the range in which the true OR lies with a 95% probability. A Χ^2^ (Chi-square) test of independence was conducted to examine the relationship between in-hospital mortality and each variable under consideration. A statistical significance level (alpha) of 0.05 was chosen; hence, a *p*-value less than this threshold would denote a statistically significant association between the factor and in-hospital mortality.

### 2.3. Ethical Considerations

The Informed Consent and Investigational Review Board (IRB) was not required for this cross-sectional study as it used data from an anonymous, de-identified, administrative database.

## 3. Results

The current study describes the secondary diagnoses of a previously published cohort of de-identified patients [20]. Briefly, the cohort of 9753 VO cases in 2020 consisted of mainly elderly patients, with 6.066 (62.6%) patients aged 70 years or older and a male/female ratio of 1.5.

In total, 150.958 secondary diagnoses were documented per 9753 cases in 2020. This results in 16 secondary diagnoses per case on average. The most common comorbidities were arterial hypertension (55.6%), type II diabetes (28.9%), and congestive heart failure (25.9%). Analysis of complications revealed hypokalemia (34.9%) and anemia due to bleeding (20.9%) followed by spinal abscess (intra- and extradural, 16.5%) to be the most documented (Table 1).

### Risk Factors for In-Hospital Mortality

**Figure 1:**
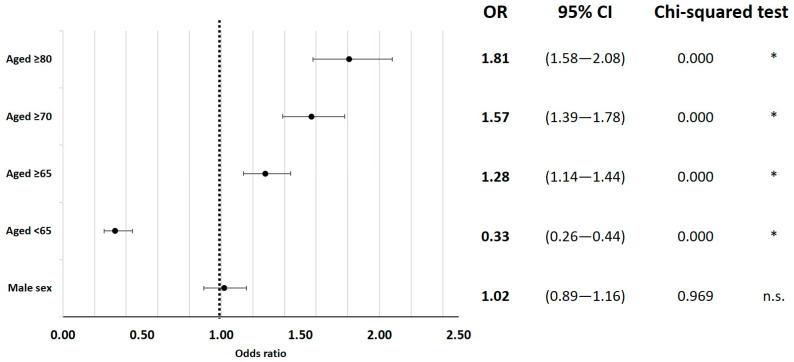
The in-hospital mortality odds ratio for epidemiological factors. * indicates *p* < 0.05.

Risk factors for in-hospital mortality were examined and several significant associations were identified. Patient age was found to have a significant impact on mortality, with higher age categories showing increased risk (65 years and older: OR = 1.28; 95% CI 1.14 to 1.44; 75 years or older: OR = 1.57; 95% CI 1.39 to 1.78; 80 years or older: OR = 1.81; 95% CI 1.58 to 2.08; all *p* < 0.01) (Figure 1). 

**Figure 2:**
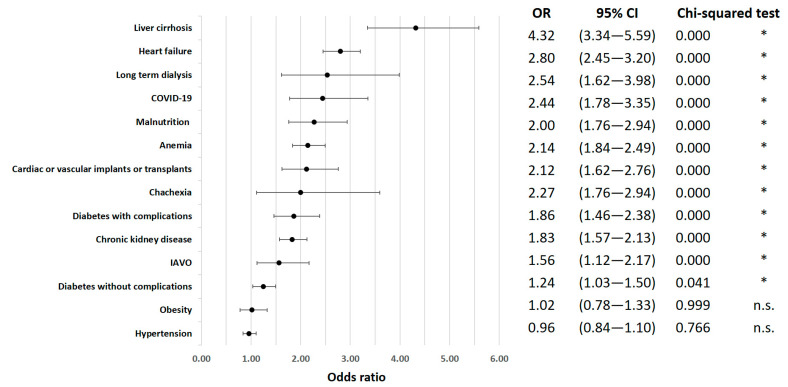
In-hospital mortality odds ratio for comorbidities. * indicates *p* < 0.05.

Specific comorbidities were also significantly correlated with inpatient mortality. Heart failure was identified as a high-risk factor (OR = 2.80; 95% CI 2.45 to 3.20; *p* < 0.01), as well as chronic kidney disease (OR = 1.83; 95% CI 1.57 to 2.13; *p* < 0.01). Diabetes with complications (OR = 1.86; 95% CI 1.46 to 2.38; *p* < 0.01) and liver failure (OR = 42.41; 95% CI 23.47 to 76.62; *p* < 0.01) were also notable risk factors for in-hospital death. Anemia showed a significant increase in mortality risk (OR = 2.14; 95% CI 1.84 to 2.49; *p* < 0.01) (Figure 2).

Interestingly, despite their relatively low prevalence (cachexia: 1.10%, malnutrition: 5.73% of all cases), cachexia (OR = 2.00; 95% CI 1.11 to 3.59; *p* = 0.123) and malnutrition (OR = 2.27; 95% CI 1.76 to 2.94; *p* < 0.01) were associated with a significantly increased risk of mortality. In contrast, obesity had a negligible effect on in-hospital mortality (OR = 1.02; 95% CI 0.78 to 1.33; *p* = 0.999).

**Figure 3:**
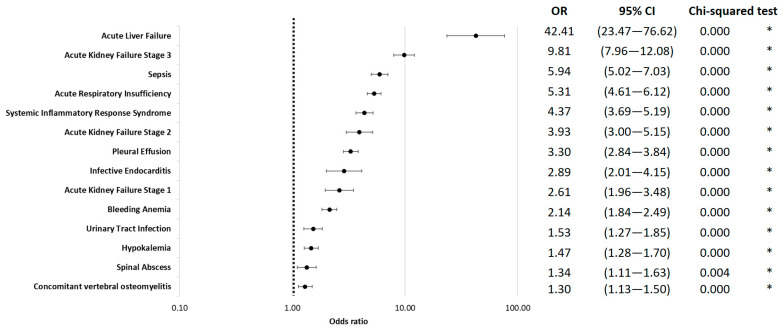
In-hospital mortality odds ratio for complications, logarithmically scaled. * indicates *p* < 0.05.

Among the complications, acute liver failure showed the highest risk for in-hospital mortality (OR = 42.41; 95% CI 23.47 to 76.62; *p* < 0.01). Additionally, stage III kidney failure (OR = 9.81; 95% CI 7.96 to 12.08; *p* < 0.01), sepsis (OR = 5.94; 95% CI 5.02 to 7.03; *p* < 0.01), acute respiratory failure (OR = 5.31; 95% CI 4.61 to 6.12; *p* < 0.01), and systemic inflammatory response syndrome (SIRS) (OR = 5.19; 95% CI 3.69 to 5.19; *p* < 0.01) were associated with in-hospital mortality (Figure 3). 

**Figure 4:**
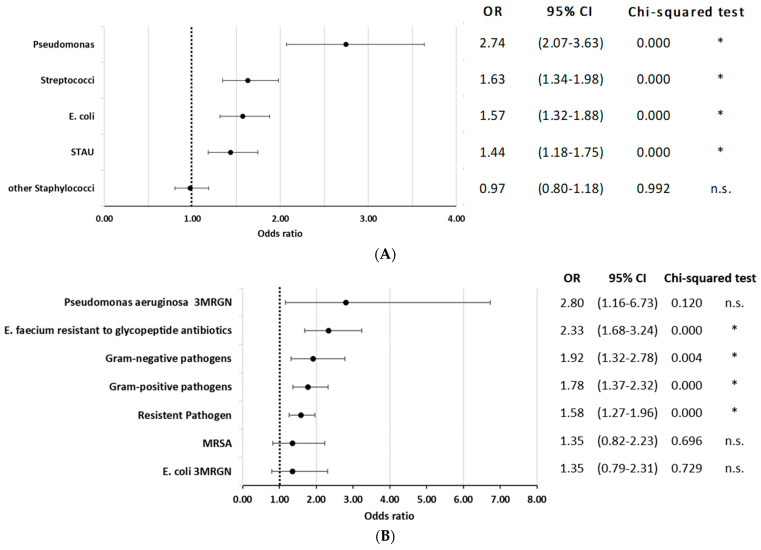
(**A**) In-hospital mortality odds ratio for coded pathogen. STAU = *S. aureus*. * indicates *p* < 0.05. (**B**) In-hospital mortality odds ratio for coded pathogen with antimicrobial resistance. * indicates *p* < 0.05.

Information about coded pathogens in the current population has been published previously [20]. Briefly, pathogens were documented in 77.8% of the cases, with “other/unspecified staphylococci”, *E. coli*, *S. aureus*, and streptococci being most prevalent. When analyzing the influence of pathogens, documented infection with *Pseudomonas aeruginosa* had the highest risk of mortality (OR = 2.74; 95% CI 2.07 to 3.63; *p* < 0.01), followed by Streptococci, *Escherichia coli*, and *Staphylococcus aureus* infections. (Figure 4A). Antimicrobial resistance codes were present in 12.9% of these cases, predominantly among gram-positive pathogens [20]. Overall, the presence of any resistant pathogen increased mortality risk (OR = 1.58; 95% CI 1.27 to 1.96; *p* < 0.000). Among gram-negative pathogens, a significant increase in mortality risk was observed (OR = 1.92; 95% CI 1.32 to 2.78; *p* = 0.004). Similarly, gram-positive pathogens also showed a significant association (OR = 1.78; 95% CI 1.37 to 2.32; *p* < 0.000). *Pseudomonas aeruginosa* with multidrug-resistance excluding carbapenems (3MRGN), although not statistically significant (OR = 2.8; 95% CI 1.16 to 6.73; *p* = 0.120), and glycopeptide antibiotic-resistant *Enterococcus faecium (E. faecium)* (OR = 2.33; 95% CI 1.68 to 3.24; *p* < 0.000) were associated with elevated mortality risk. However, *Methicillin-resistant Staphylococcus* aureus (*MRSA*) and *E. coli* 3MRGN did not show a significant impact on mortality (*p* > 0.05 for both, Figure 4B).

## 4. Discussion

### 4.1. The Risk Factors for Mortality after VO 

Associated risk factors for mortality after VO were examined in this nationwide evaluation analysis. As anticipated and previously reported, the risk of mortality increased with age, with patients aged 80 years or older having an odds ratio (OR) of 1.81 [21,22]. Additionally, secondary diagnoses contributing to frailty were associated with an increased risk. Liver cirrhosis (OR = 4.32), congestive heart failure (OR = 2.80), and the need for kidney dialysis (OR = 2.54) presented as the highest risk factors. Interestingly, obesity did not show an elevated risk in our analysis (OR = 1.02), whereas malnutrition (OR = 2.27) and cachexia (OR = 2.00) were associated with increased risk. In contrast to risk analyses reported by Vettivel et al. [21], the location or multiple levels of vertebral osteomyelitis (VO) were not identified as relevant risk factors in our study.

In a recent retrospective analysis of 155 patients with pyogenic VO, the in-hospital mortality rate was 12.9% [6]. Septic symptoms were observed in 21.9% of the patients. Yagdiran et al. reported 1- and 2-year mortality rates of 20% and 23%, respectively [23]. Vettivel et al. conducted a single-center study on 76 patients with pyogenic VO and reported a mortality rate of 5.2% at 30 days and 22.3% at 1 year. They identified the presence of frailty (OR = 13.62) and chronic renal failure (OR = 13.40) as risk factors for elevated 30-day mortality [21]. These findings closely resemble the current results, which encompassed the entirety of hospitalized patients.

Complications associated with in-hospital mortality include liver failure (OR = 42.41) and stage III acute kidney failure (OR = 9.81). Additionally, infectious complications such as endocarditis, pleural effusion, and systemic inflammatory response syndrome (SIRS) were significant factors. The presence of sepsis resulted in an odds ratio of 5.94 for in-hospital mortality. It is estimated that the global mortality rate for in-hospital-treated sepsis is approximately 25% for sepsis and 35% for severe sepsis at 30 days [24,25]. Sepsis-associated acute kidney injury is a common complication in critically ill patients and is associated with high morbidity and mortality [26,27]. Conversely, acute kidney injury has been identified as a risk factor for sepsis and its adverse outcomes [28]. Similarly, acute and acute-on-chronic liver failure have a negative impact on the prognosis of sepsis and serve as independent predictors of mortality in the intensive care unit [29,30,31]. There is limited evidence regarding organ failure as a risk factor for mortality in vertebral osteomyelitis (VO). This evaluation, to the best of our knowledge, identifies chronic and acute kidney and liver diseases and failure as significant risk factors for in-hospital mortality.

Apart from Staphylococcus aureus infections (OR = 1.44), the presence of gram-negative pathogens was associated with higher mortality. Specifically, Pseudomonas aeruginosa infection was identified as a relevant risk factor (OR = 2.74). The current results underscore the challenge that antimicrobial resistance poses in VO management. Particularly, *Pseudomonas aeruginosa* 3MRGN (OR = 2.80) and glycopeptide antibiotic-resistant *Enterococcus faecium* (OR = 2.33) showed a substantial contribution to mortality risk, stressing the importance of considering local resistance patterns in empirical antibiotic therapy for VO. These findings emphasize the need for tailored antibacterial strategies and antibiotic stewardship in tackling resistant pathogens in VO, as previously suggested [6]. In a retrospective analysis of 344 cases, Kang et al. found that patients with pyogenic spondylitis caused by gram-negative pathogens had a higher frequency of severe sepsis (24.2% vs. 11.3%), but the mortality rate did not significantly differ compared to infections with gram-positive pathogens (6.0% vs. 5.2%) [32].

Our results suggest that VO patients with chronic organ diseases should be closely monitored, and early detection and treatment of sepsis are crucial [33]. One possible approach could be the implementation of broad empirical antibiotic treatment tailored to local pathogens in this at-risk group, including cases of culture-negative VO. Identifying the specific pathogen and conducting antibiotic susceptibility testing are essential and should be pursued in all cases [34,35].

### 4.2. Strengths and Limitations

One notable strength of this epidemiological evaluation is its utilization of registry data comprising ICD-10 diagnosis codes from all medical institutions in Germany. The availability of these datasets facilitated a univariate analysis of risk factors for in-hospital mortality rates. The findings of this study should be interpreted within the scope of its limitations. In general, registry studies are commonly not accepted by design to allow causative relationships to be made. The limitations of our study include the inherent challenges of coding, which constrains the precise identification of certain pathogens, beyond the given ICD-10 codes. Further, the assessment of one-year or long-term mortality was not feasible. It is important to note that only inpatient data were accessible, and the detailed analysis focused solely on primary diagnoses. Additionally, this analysis does not provide information on treatment modalities such as surgery or antimicrobial therapy, thus precluding their consideration in the risk analysis. A significant limitation of this study is the lack of information regarding the administration of antimicrobials to patients. The ICD-10-based dataset does not capture specific therapeutic interventions, including antimicrobial use. This constraint precludes the assessment of the potential impact of antimicrobial administration on patient outcomes. Therefore, it is important to carefully interpret the data and consider alternative approaches to such prospective studies to gain a deeper understanding of the association between the tested risk factors and in-hospital mortality.

## 5. Conclusions

This analysis identified several comorbidities and complications as potential risk factors for elevated in-hospital mortality in pyogenic VO cases on a national scale. Advanced age, secondary diagnoses contributing to frailty, and specific comorbidities such as liver cirrhosis, congestive heart failure, and the need for kidney dialysis were identified as significant risk factors. Notably, obesity did not show an increased risk, while malnutrition and cachexia were associated with higher mortality rates. The presence of sepsis, gram-negative pathogens, particularly *Pseudomonas aeruginosa* infection, and acute kidney and liver failure were also associated with increased mortality. These findings emphasize the importance of closely monitoring VO patients with chronic organ diseases, early detection and treatment of sepsis, and tailored empirical antibiotic therapy. The identification of specific pathogens and antibiotic susceptibility testing should be prioritized to improve patient outcomes in this high-risk population, mainly geriatric patients.

## Figures and Tables

**Table 1:** Most common secondary diagnoses for comorbidities and complications in VO cases: Total numbers and share of all cases in 2020.

	Secondary Diagnosis	ICD-10 Code	[n]	Percentage of All Cases
Comorbidity	Arterial Hypertension	I10.-	5424	55.6%
	Type II diabetes	E11.-	2819	28.9%
	Congestive heart failure	I50.-	2522	25.9%
	Atrial fibrillation	I48.-	2365	24.3%
	Chronic kidney disease	N18.1-; N19	2325	23.8%
	Coronary arterial disease	I25.0; I25.10-.19	1655	17.0%
	Hypothyreosis	E03.8; -.9	1264	13.0%
	Adipositas	E66.0-.99	997	10.2%
	Malignancy	C01; C10.–C97	736	7.6%
	Malnutrition	E43–E46	559	5.7%
	Implant-associated vertebral osteomyelitis	T81.4	431	4.4%
	Liver cirrhosis	K74.6; .-70-72	365	3.7%
	Dialysis	Z99.2	154	1.6%
	Cachexia	R64	107	1.1%
Complications	Hypokalemia	E87.6	3408	34.9%
	Anemia, bleeding	D62	2039	20.9%
	Spinal abscess	G06.1; -.2	1612	16.5%
	Urinal tract infection	N39.0	1595	16.4%
	Pleural infusion	J90; J91	1576	16.2%
	Acute respiratory insufficiency	J96.01; J96.00	1441	14.8%
	Acute kidney failure	N17.0-; N17.81-3, -9-; N17.91-3, -9	1185	12.2%
	Pneumonia	J12.8–J18.9	1066	10.9%
	Infectious myositis	M60.05	1010	10.4%
	SIRS	R65.0-.3	980	10.1%
	Sepsis	A40.1–8; A41.1–9	854	8.8%
	COVID-19	U07.1, .2	332	3.4%
	Infective Endocarditis	I33.0	222	2.3%
	Acute liver failure	K72.0	59	0.6%

## Data Availability

All data presented in this study are available on request from the corresponding authors.
